# Acute Chagas Disease Manifesting as Orbital Cellulitis, Texas, USA

**DOI:** 10.3201/eid2711.203698

**Published:** 2021-11

**Authors:** F. Parker Hudson, Natalie Homer, Aliza Epstein, Kristin Mondy

**Affiliations:** University of Texas Austin Dell Medical School, Austin, Texas, USA (F.P. Hudson, K. Mondy);; Texas Oculoplastics Consultants, Austin (N. Homer, A. Epstein)

**Keywords:** acute Chagas disease, triatomine, orbital cellulitis, next-generation sequencing, vector-borne infections, *Trypanosoma cruzi*, Texas, parasites

## Abstract

We report a case of acute, vectorborne Chagas disease, acquired locally in central Texas, USA, manifesting as Romaña’s sign, which was initially mistaken for orbital cellulitis. After the infection failed to respond to antibiotics, DNA-based next generation sequencing on plasma yielded high levels of *Trypanasoma cruzi*; results were confirmed by PCR.

Infection with *Trypanosoma cruzi*, or Chagas disease, is endemic in Latin America. An estimated >300,000 cases of chronic Chagas disease exist in the United States, predominantly in immigrants who acquired it through vectorborne transmission from triatomine insects in their countries of origin (*1*). Multiple triatomine species also exist in the southern United States, and enzootic transmission to dogs or small rodents is common (*1*–*3*). However, local cases of vectorborne *T. cruzi* transmission to humans is rare. In a 2009 review, the number of acute, autochthonous, vectorborne infections acquired in the United States since 1955 was only 7; of these, 4 occurred in Texas (*4*). During 2013–2018, a total of 26 locally acquired Chagas cases were reported in Texas, but only 1 was an acute case (*5*). Most persons with acute Chagas are asymptomatic, but some have nonspecific constitutional symptoms or Romaña’s sign, a painless periorbital and conjunctival injection attributed to triatomine feces deposited or inadvertently rubbed into the eye. 

Acute Chagas disease may be overlooked in areas outside of Latin America, despite the existence of *T.* c*ruzi*–infected triatomine reservoirs (*6*). We describe a case of locally acquired, vectorborne, acute Chagas disease in a patient who manifested Romaña’s sign; the infection was initially mistaken for orbital cellulitis. We also describe the use of next-generation sequencing (NGS) as a helpful diagnostic tool and review potential vector transmission risk in the southern United States.

## The Patient 

**Figure 1:**
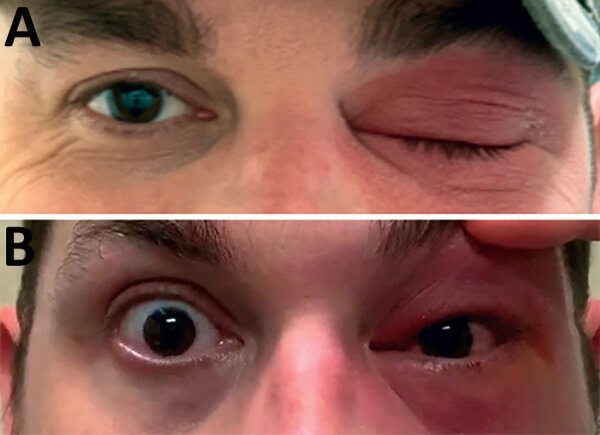
Patient with acute Chagas disease manifesting as orbital cellulitis, Texas, USA, on the day he first accessed care. A) Left periorbital edema and erythema. B) Conjunctival injection.

A previously healthy 41-year-old man from central Texas with no medical or travel history described an ocular foreign-body sensation after working outdoors at his ranch. Over the next 2 days he experienced mild conjunctival injection with periorbital erythema and edema (Figure 1). He described mildly blurry vision but no fever, chills, or constitutional symptoms. On day 3 of illness, his primary care provider prescribed oral trimethoprim/sulfamethoxazole and ophthalmic ciprofloxacin drops. The patient’s eye symptoms worsened, and on day 5 of illness an ophthalmologist hospitalized him for orbital cellulitis. The patient had normal visual acuity. A computed tomography scan demonstrated nonspecific preseptal and postseptal inflammatory changes, consistent with mild orbital cellulitis. The patient was initiated on intravenous vancomycin and piperacillin/tazobactam. 

Results of testing for HIV, syphilis, and methicillin-resistant *Staphylococcus aureus* nasal colonization were negative. On the eighth day of illness, the patient had a new palpable, left preauricular lymph node. Given the suspicion for oculoglandular syndrome, the patient was switched to intravenous rifampin and doxycycline for possible *Bartonella* infection. However, results of *Bartonella* serologic testing and blood cultures were negative. 

On day 11 of illness, the patient had onset of fever. His periorbital edema and lymphadenopathy persisted. His antibiotics were broadened to include vancomycin, cefepime, and acyclovir. He remained febrile, and on day 15 he was transferred to a tertiary care center for evaluation by infectious diseases, oculoplastic surgery, and rheumatology specialists. Further history revealed that he frequently stayed at a local ranch with horses and small mammals, including rabbits, squirrels, and stray cats. He otherwise resided at his suburban residence with his family and two dogs and worked indoors at a nearby university. A magnetic resonance imaging scan demonstrated persistent enhancement of left medial and inferior rectus muscles, compatible with orbital myositis. However, the patient never experienced pain or limitations of extraocular movements. Results of additional tests for toxoplasmosis, tularemia, and adenovirus were negative. 

**Figure 2:**
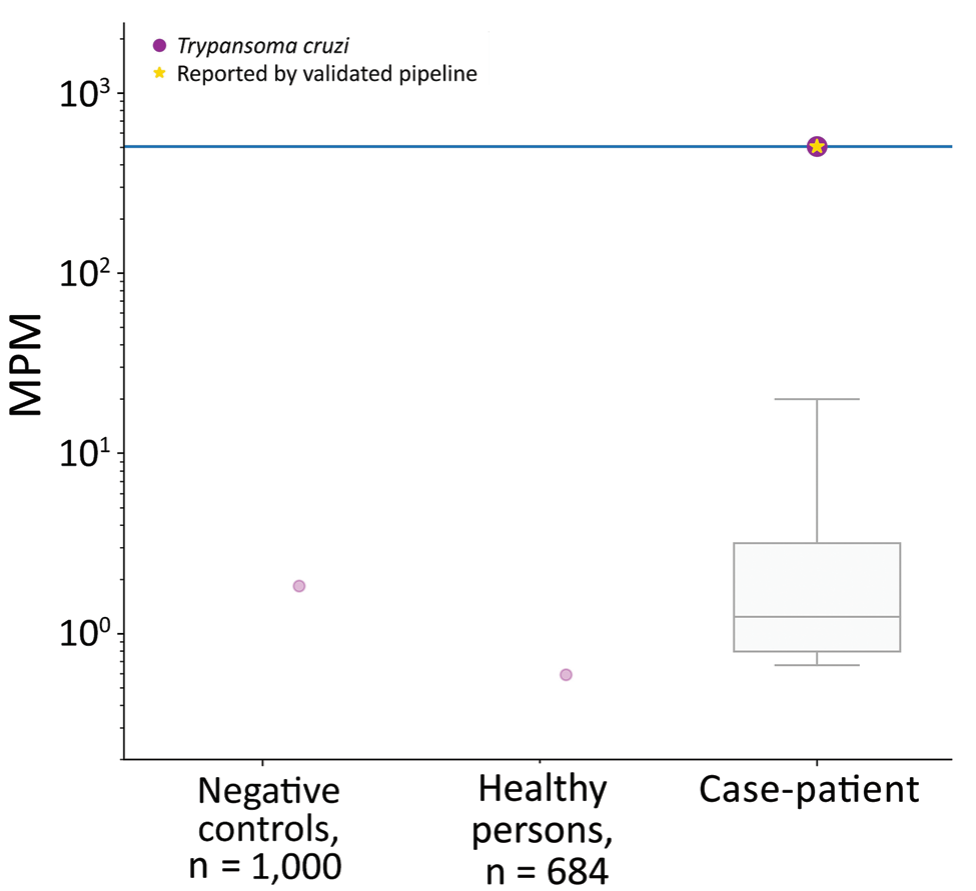
Results of next-generation sequencing on a plasma sample of a patient with acute Chagas disease manifesting as orbital cellulitis, Texas, USA, showing the very high detection of *Trypanosoma cruzi* (505.61 MPM) (right). The comparison with 1,000 aggregated negative controls (buffer/reagents) show a very low microbial cell-free DNA (mcfDNA) signal, which aligned to *T. cruzi* in 1 sample (left). A cohort of 684 healthy controls had a very low mcfDNA signal, which aligned to *T. cruzi* in 1 person (middle). The purple dots indicate any mcfDNA that align to *T. cruzi*; the gold star indicates an mcfDNA detection that represents a positive result for *T. cruzi* identified by the Karius Test (Karius, Inc., https://kariusdx.com). The other detected but not clinically or statistically significant mcfDNA in the case patient’s sample are also shown in the gray box and whisker plot on the log scale; horizontal line within box indicates median, box top and bottom indicate interquartile range (Q1–Q3), and error bars indicate range. MPM, molecules/μL.

We sent plasma to Karius, Inc. (https://kariusdx.com; Redwood City, California, USA), to undergo the Karius Test, which uses next-generation sequencing to detect foreign pathogens. On day 16, the patient was started on steroids for possible noninfectious etiologies, including IgG4-related disease, sarcoidosis, or vasculitis. His fever subsequently abated, and the periorbital edema and erythema slightly improved. On day 19, he was discharged on a tapering course of oral steroids and an additional 7 days of oral antibiotics. After discharge, results of the Karius Test were positive for *T. cruzi* at a high level of 505 DNA molecules/μL (Figure 2). Additional testing through the Texas Department of State Health Services and the Centers for Disease Control and Prevention yielded a positive *T. cruzi* PCR test, confirming acute infection. The patient received benznidazole (200 mg 2×/d for 60 d), and steroids were discontinued. He reported some initial general malaise during the first week of therapy but thereafter had only mild generalized itchiness but no rash. He had complete resolution of clinical disease by the fifth week of treatment.

## Conclusions

Human acquisition of Chagas disease in the United States remains rare, but this rarity may be attributable in part to the protean manifestations of acute disease, resulting in missed diagnoses. The World Health Organization estimates that <50% of symptomatic persons with acute infection will have a visible sign of a triatomine bite (chagoma) or the classic Romaña’s sign (*7*). We found NGS testing to be useful in diagnosing acute Chagas. This patient continued to have ocular manifestations and fever despite broad-spectrum antibiotics and an extensive work-up for the broad differential of oculoglandular diseases. 

The Karius Test is a commercially available NGS test for which detailed methodology has been previously described (*8*,*9*). The Karius laboratory extracts and subsequently sequences microbial cell-free DNA from a plasma sample. Within 48 hours, the test indicates bacteria, fungi, DNA viruses, and parasites present at levels greater than a predefined threshold, after removal of human sequences. NGS testing may be a particularly useful tool in atypical febrile syndromes such as in this case, where a very broad range of differential diagnoses were considered (*8*). Such cases require numerous serologic tests, some of which may have variable sensitivity and specificity, costs, and delays in diagnosis and hospital discharge. Otherwise, acute Chagas is typically diagnosed by either identifying the parasite on a blood smear or sending a blood sample to the Centers for Disease Control and Prevention for PCR testing. Such tests would probably not be ordered outside of a known endemic area. 

Our case suggests that the extent of domestic-transmission cycles between triatomine vectors and humans is underrecognized in southern US states. In endemic regions, the primary risk for insect-to-human transmission has been related to efficiency with which local vector species can invade and colonize homes (typically in rural areas with impoverished housing conditions such as adobe, wood, and thatch) (*1*). In a study conducted in south Texas, however, large infestations of triatomine insects were found under solid foundations, including cement patios connected to houses and car garages, and >50% of the insects were infected with *T. cruzi* (*2*). In another study of multiple residential sites in central Texas, a high proportion of the triatomine specimens found were infected *T. cruzi*, including 69% of those found inside houses, 81% of those found outside houses, and 82% of those found in dog kennels (*3*). Finally, prolonged periods outdoors, especially while hunting and camping, have been hypothesized to contribute to transmission (*10*). Although the vector and pathogen are quite ubiquitous, the contact between vectors and humans while sleeping and the inefficiency of transmission may account for lower rates of acute infection in Texas compared with Latin America.

In light of our findings, physicians need to be aware of the risk for vectorborne transmission of Chagas disease in and around residential areas, particularly in southern areas of the United States such as Texas. Prompt recognition and treatment of acute Chagas can lead to cure as well as prevention of the illness and risk for death associated with chronic Chagas disease, unnecessary hospitalization, or worsening of the patient’s condition from the use of immunosuppressive agents.

## References

[1] Bern C, Kjos S, Yabsley MJ, Montgomery SP. *Trypanosoma cruzi* and Chagas’ disease in the United States. Clin Microbiol Rev. 2011;24:655–81. 10.1128/CMR.00005-11 21976603 PMC3194829

[2] Beard CB, Pye G, Steurer FJ, Rodriguez R, Campman R, Peterson AT, et al. Chagas disease in a domestic transmission cycle, southern Texas, USA. Emerg Infect Dis. 2003;9:103–5. 10.3201/eid0901.020217 12533289 PMC2873735

[3] Kjos SA, Marcet PL, Yabsley MJ, Kitron U, Snowden KF, Logan KS, et al. Identification of bloodmeal sources and *Trypanosoma cruzi* infection in triatomine bugs (Hemiptera: Reduviidae) from residential settings in Texas, the United States. J Med Entomol. 2013;50:1126–39. 10.1603/ME12242 24180119 PMC3932564

[4] Bern C, Montgomery SP. An estimate of the burden of Chagas disease in the United States. Clin Infect Dis. 2009;49:e52–4. 10.1086/605091 19640226

[5] Texas Department of State Health Services. Chagas disease data [cited 2020 Apr 28]. https://www.dshs.texas.gov/IDCU/disease/chagas/Chagas-Disease-Data.aspx

[6] Garcia MN, Woc-Colburn L, Aguilar D, Hotez PJ, Murray KO. Historical perspectives on the epidemiology of human Chagas disease in Texas and recommendations for enhanced understanding of clinical Chagas disease in the southern United States. PLoS Negl Trop Dis. 2015;9:e0003981. 10.1371/journal.pntd.0003981 26540273 PMC4634991

[7] World Health Organization. Chagas disease [cited 2020 Apr 28] https://www.who.int/chagas/disease/home_symptoms_more

[8] Rossoff J, Chaudhury S, Soneji M, Patel SJ, Kwon S, Armstrong A, et al. Noninvasive diagnosis of infection using plasma next-generation sequencing: a single-center experience. Open Forum Infect Dis. 2019;6:1–3. 10.1093/ofid/ofz327 31375834 PMC6677669

[9] Vudatha V, Ranson M, Blair L, Ahmed AA. Rapid detection of bacille Calmette-Guérin-associated mycotic aortic aneurysm using novel cell-free DNA assay. J Vasc Surg Cases Innov Tech. 2019;5:143–8. 10.1016/j.jvscit.2018.11.006 31193416 PMC6529677

[10] Garcia MN, Hotez PJ, Murray KO. Potential novel risk factors for autochthonous and sylvatic transmission of human Chagas disease in the United States. Parasit Vectors. 2014;7:311. 10.1186/1756-3305-7-311 24996479 PMC4094476

